# A novel risk score for disease control prediction of chronic rhinosinusitis

**DOI:** 10.1111/coa.13949

**Published:** 2022-06-30

**Authors:** Lijie Jiang, Kanghua Wang, Tengjiao Lin, Yifeng Jiang, Wenxiang Gao, Cong Li, Zhaoqi Huang, Chuxin Chen, Zhiyin Nie, Rui Zheng, Yueqi Sun, Jianbo Shi, Yinyan Lai

**Affiliations:** ^1^ Otorhinolaryngology Hospital, The First Affiliated Hospital Sun Yat‐sen University Guangzhou China; ^2^ Department of Otorhinolaryngology, The Seventh Affiliated Hospital Sun Yat‐sen University Shenzhen China; ^3^ Department of Radiation Oncology The First Affiliated Hospital of Guangzhou University of Chinese Medicine Guangzhou China; ^4^ Department of Otorhinolaryngology, The Third Affiliated Hospital Sun Yat‐sen University Guangzhou China

**Keywords:** chronic rhinosinusitis, disease control, model, prognosis

## Abstract

**Objectives:**

To assess the impact of risk factors on the disease control among chronic rhinosinusitis (CRS) patients, following 1 year of functional endoscopic sinus surgery (FESS), and combining the risk factors to formulate a convenient, visualised prediction model.

**Design:**

A retrospective and nonconcurrent cohort study.

**Setting and Participants:**

A total of 325 patients with CRS from June 2018 to July 2020 at the First Affiliated Hospital of Sun Yat‐sen University, the Third Affliated Hospital of Sun Yat‐sen University, the Seventh Affiliated Hospital of Sun Yat‐sen University.

**Main Outcomes Measures:**

Outcomes were time to event measures: the disease control of CRS after surgery 1 year. The presence of nasal polyps, smoking habits, allergic rhinitis (AR), the ratio of tissue eosinophil (TER) and peripheral blood eosinophil count (PBEC) and asthma was assessed. The logistic regression models were used to conduct multivariate and univariate analyses. Asthma, TER, AR, PBEC were also included in the nomogram. The calibration curve and area under curve (AUC) were used to evaluate the forecast performance of the model.

**Results:**

In univariate analyses, most of the covariates had significant associations with the endpoints, except for age, gender and smoking. The nomogram showed the highest accuracy with an AUC of 0.760 (95% CI, 0.688–0.830) in the training cohort.

**Conclusions:**

In this cohort study that included the asthma, AR, TER, PBEC, which had significantly affected the disease control of CRS after surgery. The model provided relatively accurate prediction in the disease control of CRS after FESS and served as a visualised reference for daily diagnosis and treatment.

**Key Points**

1. What is the risk factor affecting the disease control of CRS after surgery and which way is the most accuracy to predict the prognosis?
2. Our study assessed the impact of risk factors on the disease control among CRS patients, following 1 year of functional endoscopic sinus surgery (FESS).
3. Asthma and the ratio of tissue eosinophil are the most important risk factor affected the disease control of CRS.
4. Our study combined the risk factors to formulate a convenient, visualised prediction model.
5. This study also had some limitations due to the small cohort size, lead to the inspection efficiency.

## 1 INTRODUCTION

Chronic rhinosinusitis (CRS) is a multifactorial heterogeneous disease, although its pathogenesis and precise mechanism remains largely unclear. Due to the poor understanding of the pathophysiology of CRS, it affects the quality of life of patients and increases the cost burden as compared to people without CRS. It is estimated to affect 8% of the adult population in China.^1^ According to the EPOS2020, the current treatment for CRS includes medical therapy and FESS with the final target to achieve cure or clinical control.^2^ Although, the disease state of more than 30% of patients with nasal polyps, remains uncontrolled despite the current medical therapy (AMT) and FESS.^3^ DeConde et al. also reported the disease relapse in 40% of patients with nasal polyps after 18 months.^4^ The latest evidence has further indicated that the underlying diversity of endotypes might be a crucial reason for the unconformity in clinical phenotype and disease prognosis.^5^ Therefore, it is essential to find relevant clinical markers and to make a convenient model to predict the poor disease control in CRS.

Emerging evidence has proven that eosinophil (EOS) inflammation is a dominant factor associated with CRS recurrence and poor disease control.^6^ In addition to the local eosinophils, peripheral blood eosinophils are also associated with CRS and can be a reliable marker for predicting the prognosis of CRS. Some studies have demonstrated the peripheral blood eosinophil as a marker for the EOS CRS.^6^ In a recent study, Guiherme et al., suggested that asthma was a dominant factor for the recurrence of CRS.^7^ Nonetheless, some studies have reported that inhalant allergens may lead to poor sinus CT and endoscopic scores. But several studies have found no difference in allergic and nonatopic patients on the sinusitis severity.^8^ Thus, it is deemed necessary to evaluate the role of allergy in nasal polyps' disease control.

Undeniably studies on predictive factors of CRS treatment outcomes are crucial and can help improvise personalised and integration management of CRS in various hospitals. Therefore, this study aims to evaluate the risk factors involved in the prognosis of CRS after 1 year of undergoing endoscopic sinus surgery and combined the risk factors to establish a convenient and accurate prediction model.

## 2 MATERIAL AND METHODS

This is a retrospective and nonconcurrent cohort study. The study was approved by the local Ethics Committee ([2017]164). According to the European Position Paper on Rhinosinusitis and Nasal Polyps 2012 (EPOS2012) guidelines, patients who satisfied the diagnostic criteria of CRS with nasal polyps were included in the study from the First Affiliated Hospital of Sun Yat‐sen University, the Third Affliated Hospital of Sun Yat‐sen University, the Seventh Affiliated Hospital of Sun Yat‐sen University. All patients received FESS between June 2018 to July 2020 and were periodically reassessed during their routine outpatient visits following the surgery. These patients were initially treated with AMT i.e., nasal steroids (drops/sprays/rinses), saline rinses, educated regarding technique, oral corticosteroid short‐course (OCS) and two‐course antibiotics before surgery.

The enrolled participants according to the following inclusion criteria: (1) Age ≥18 years; (2) CRSwNP was diagnosed based on the European position paper on rhinosinusitis and nasal polyps (EPOS 2012); (3) Patients were performed bilateral endoscopic sinus surgery; (4) LM CT unilateral score >6. In addition, patients with following criteria were excluded: (1) Patients without complete data of baseline blood routine test, tissue specimens, sinus computed tomography and nasal endoscopy; (2) Patients prescribed with systemic or intranasal corticosteroids within 1 month before blood routine test, by cross‐referencing patient's medication history with the electronic prescription record system of the hospital. (3) Patients were younger than 18 years of age. (4) Patients with a history of allergic dermatitis, food allergies or helminth infection. (5) Patients with cystic fifibrosis, fungal rhinosinusitis, sinonasal malignancies.

Patients were instructed to use topical corticosteroids‐budesonide nasal spray (256 μg/day for 6 months), and intranasal budesonide suspension (1 mg/day for 4 weeks) after surgery. They were reassessed periodically at their routine outpatient visits at 1–3 months after surgery then once in 3 months until 1 year follows up. During the assessment in the follow‐up visits, if their symptoms or endoscopic signs persisted, they received new AMT, that is, nasal steroids (drops/spray/rinses), saline rinses, education regarding technique, OCS and optional two‐course antibiotics. The symptoms, endoscopic scores and modified treatment (if any) were recorded by clinicians after 1 year.

Items recorded from the enrolled patients were as following:

- Nasal symptoms;
- Lund and Kennedy score recorded by nasal endoscopy findings;
- Comorbidities: smoking habit, asthma (based on the spirometry and clinical parameters);
- Respiratory allergens;
- Peripheral blood eosinophil count before the initiation of oral corticosteroids. More than 0.3 × 10^9^/L was considered as high blood eosinophilia in CRS.

### 2.1 Data collection

Patients were divided into two groups of controlled (included partly controlled) and uncontrolled CRS, based on the disease control criteria of EPOS2020. Patients were followed up for 1 year after surgery, until the end of the study period (30th December 2020). Time‐to‐event was defined as the time starting from surgery till the 12th month post‐operatively. According to the EPOS2020, the control criteria of the CRS can be divided into symptoms, nasal endoscopy, the need for recuse treatment. Symptom substituted by ‘VAS (Visual Analogue Scale) < 5’, and ‘present/impaired’ by ‘VAS ≥5’. Furthermore, the detailed symptoms related to CRS are included in supplement Table S1. The evaluation endpoint was 12th month post‐operatively.

### 2.2 Nomogram development

The nomogram model was formulated by the results of multivariate analysis. Univariate analysis with a significant difference at *p*‐value (<.05) between all variables was included in the multivariate analysis. The *p*‐value <.05 in multivariate analysis was also included as the prognostic factor in the nomogram. Allergic rhinitis (AR) and peripheral blood eosinophil count (PBEC) were statistically significant in univariate analysis for 1 year disease control but not significant difference in multivariate analysis for 1 year disease control. However, AR and PBEC have long been recognised to determine the prognosis of CRS. AR and PBEC were also included in the nomogram for the current study, since excluding these covariates would have over‐inflated the effects of the remaining factors and decrease the predictive power of our model. The Cox proportional hazard model was used to produce nomograms for predicting the risk of the uncontrolled incident after the surgery. A score based on regression coefficients was assigned to these factors.

### 2.3 Model evaluation

The nomogram's forecast performance was evaluated by the receiver operating characteristic (ROC), the area under curve (AUC) for both training and validation cohort. In a logistic regression model, the value of AUC is the same as that yielded by the concordance index (c‐index), with values ranging from 0.5 (no predictive value) to 1.0 (complete discrimination). A larger AUC value represents a more accurate prediction of the uncontrolled disease possibility. The agreement between the predicted uncontrolled incident and the observed uncontrolled incident after bias correction was quantified by the calibration curves of the nomogram for determining the uncontrolled incident rate. Decision curve analysis (DCA) was also carried out to compare the potential net benefit of the predictive models.

### 2.4 Statistical analysis

We compared the patient pathologic characteristics and demographic profile between training and validation cohort by using Fisher's exact tests and chi‐squared tests. Multivariate logistic regression analysis was used to distinguish the independent risk factors associated with uncontrolled disease. Nomogram development was carried out by using the library ‘rms’ in R for MACOS. All statistical analyses were conducted by the R software version 4.0.2 (R Foundation for Statistical Computing, Vienna, Austria; www.R-project.org). The *p* values <.05 were considered statistically significant.

## 3 RESULTS

A total of 397 patients with CRS with nasal polyps (CRSwNP) from June 2018 to July 2020 at the First Affiliated Hospital of Sun Yat‐sen University, the Third Affliated Hospital of Sun Yat‐sen University, the Seventh Affiliated Hospital of Sun Yat‐sen University. We included 325 patients who were following the doctor's instructions and had a follow‐up for 1 year. The enrolled patients were randomly assigned to a training (*n* = 195) and validation cohort (*n* = 130). The nomogram was based on the training cohort and its accuracy was internally validated through the validation cohort. The baseline characteristics of the CRS patients between the training cohort and validation cohort are shown in Table 1. No significant differences were observed for these characteristics between the training and validation cohort. Univariate analyses were done with the primary objective to confirm the statistical effect between each covariate and the endpoints. Results showed that most covariates had statistically significant associations with the endpoints, except for age, gender and smoking (Table 2).

**TABLE 1:** Demographic and clinicopathological characteristics of patients with chronic rhinosinusitis

Characteristics	Training cohort (*N* = 195) (%)	Validation cohort (*N* = 130) (%)	*p* value
Age (median [range])	44.00 [17, 74]	40.00 [16.00, 74.00]	.065
Preoperative_LK_score (median [range])	10.00 [3.00, 15.00]	10.00 [4.00, 12.00]	.693
Lund Mackay score (median [range])	17.00 [0.00, 28.00]	16.50 [2.00, 27.00]	.626
Gender			.981
Male	120 (61.5)	89 (60.8)	
Female	75 (38.5)	51 (39.2)	
Smoking			.778
No	177 (90.8)	120 (92.3)	
Yes	18 (9.2)	10 (7.7)	
AR			.087
No	152 (77.9)	112 (86.2)	
Yes	43 (22.1)	18 (13.8)	
Asthma			.133
No	121 (62.1)	92 (70.8)	
Yes	74 (37.9)	38 (29.2)	
Blood eosinophil number			.374
<0.3	113 (57.9)	68 (52.3)	
≥0.3	82 (42.1)	62 (47.7)	
Tissue eosinophil ratio			.910
<10	95 (48.7)	65 (50.0)	
≥10	100 (51.3)	65 (50.0)	
Tissue eosinophil number			.872
<10	81 (41.5)	56 (43.1)	
≥10	114 (58.5)	74 (56.9)	

**TABLE 2:** Univariate and multivariable logistic regression analyses reporting the odds ratios (ORs) for risk of uncontrolled in the training cohort

Variable	Univariate analysis		Multivariate analysis	
	OR (95%)	*p*	HR (95%)	*p*
Age	1.013 (0.9905–1.037)	.288	NI	
Preoperative LK scoring	1.174 (1.014–1.371)	.037	1.145 (0.958–1.380)	.143
Lund Mackay score	1.058 (1.000–1.123)	.001	0.967 (0.889–1.039)	.338
Gender				
Male	Ref		NI	
Female	1.079 (0.579–1.991)	.809	NI	
Smoking				
No	Ref		NI	
Yes	0.789 (0.244–2.203)	.667		
AR				
No	Ref		Ref	
Yes	2.836 (1.413–5.732)	.003	1.294 (0.568–2.903)	.533
Asthma				
No	Ref		Ref	
Yes	4.053 (2.168–7.725)	<.001	2.558 (1.154–5.763)	.021
Blood eosinophil number				
<0.3	Ref		Ref	
≥0.3	3.727 (2.002–7.087)	<.001	2.029 (0.924–4.489)	.078
Tissue eosinophil number				
<10	Ref		NI	
≥10	4.923 (2.552–9.944)	<.001	1.028 (0.422–2.454)	.951
Tissue eosinophil ratio				
<10	Ref		Ref	
≥10	2.051 (1.096–3.942)	.002	2.947 (1.284–7.008)	.012

Abbreviations: HR, hazard ratio; NI, not include; OR, odds ratio; Ref, reference.

### 3.1 Nomogram development

**FIGURE 1:**
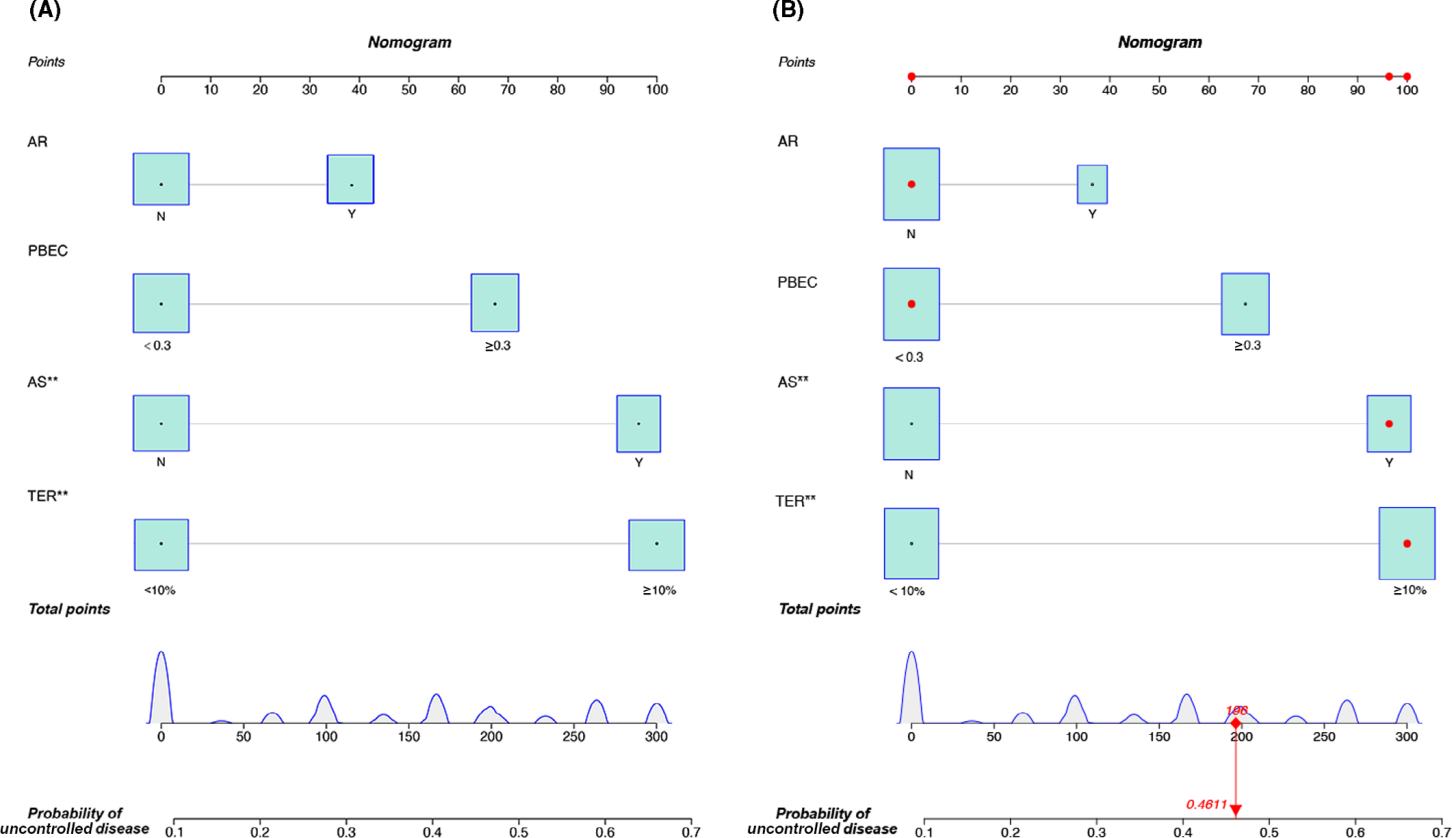
Postoperative nomogram predicting 1‐year probability of uncontrol disease after endoscopic surgery. (A) Each clinical variable has a certain number of points (top row) ranging from 0 to 100. The sum of points of each variable was related to the probability of uncontrol disease at 1 year. (B) An example illustrating the use of the nomogram. This patient was one of the training cohort in the current study. The patient has tissue eosinophil ratio ≥10% (points = 100), low blood eosinophilia (points = 0), no AR (points = 0) and asthma (points = 96), thus the total points are 196 and the corresponding risk event of recurrence is 46.11%. AS, asthma; PBEC, peripheral blood eosinophil count; TEN, tissue eosinophil number; TER, tissue eosinophil ratio

After the initial univariate analyses with extensive review of the medical literature, we included all the covariates in the subsequent multivariate logistic regression models, except for age, gender, smoking, tissue eosinophil counts, preoperative Lund Kennedy score and Lund Mackay score. Based on these factors, the nomogram was constructed for calculating the risk of recurrence of the CRS after operation 1 year (Figure 1A).

A case demonstrating our nomogram usage is shown in Figure 1B. For example, if the patient had tissue eosinophil ratio ≥10%, low blood eosinophilia, no AR and asthma, then the total points would be 196 with the corresponding risk of recurrence at 46.11%.

### 3.2 Nomogram validation

**FIGURE 2:**
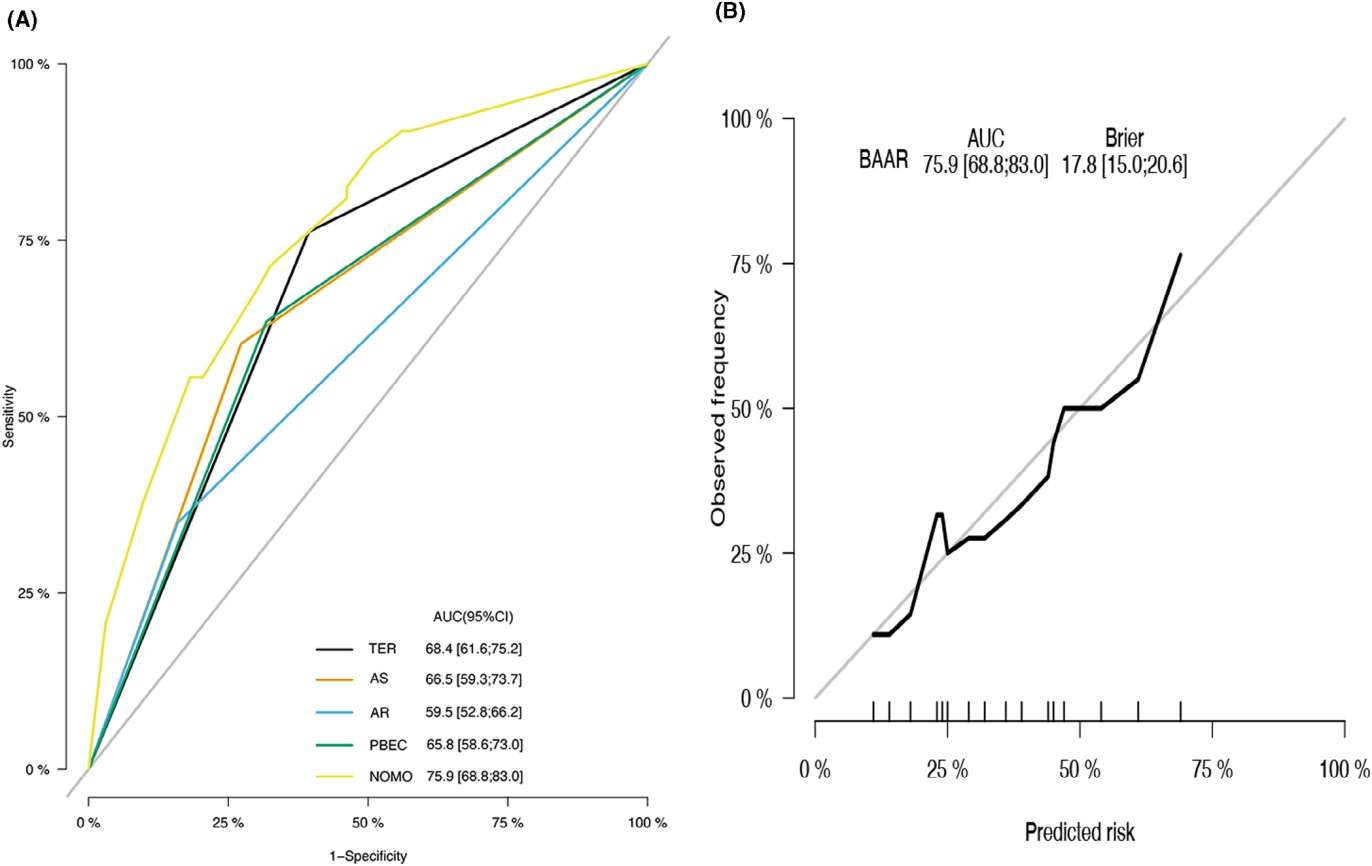
(A) ROC curves of the training cohort predicting 1‐year probability of uncontrol disease after endoscopic surgery with corresponding AUC values. (B) Calibration in the primary cohort for predicting patient risk of recurrence. The x‐axis is nomogram‐predicted probability of survival and y‐axis is actual survival. The reference line is 45° and indicates perfect calibration. AS, asthma; AUC, area under curve; CI, confidence interval; PBEC, peripheral blood eosinophil count; ROC, receiver operating characteristic; TEN, tissue eosinophil number; TER, tissue eosinophil ratio

**FIGURE 3:**
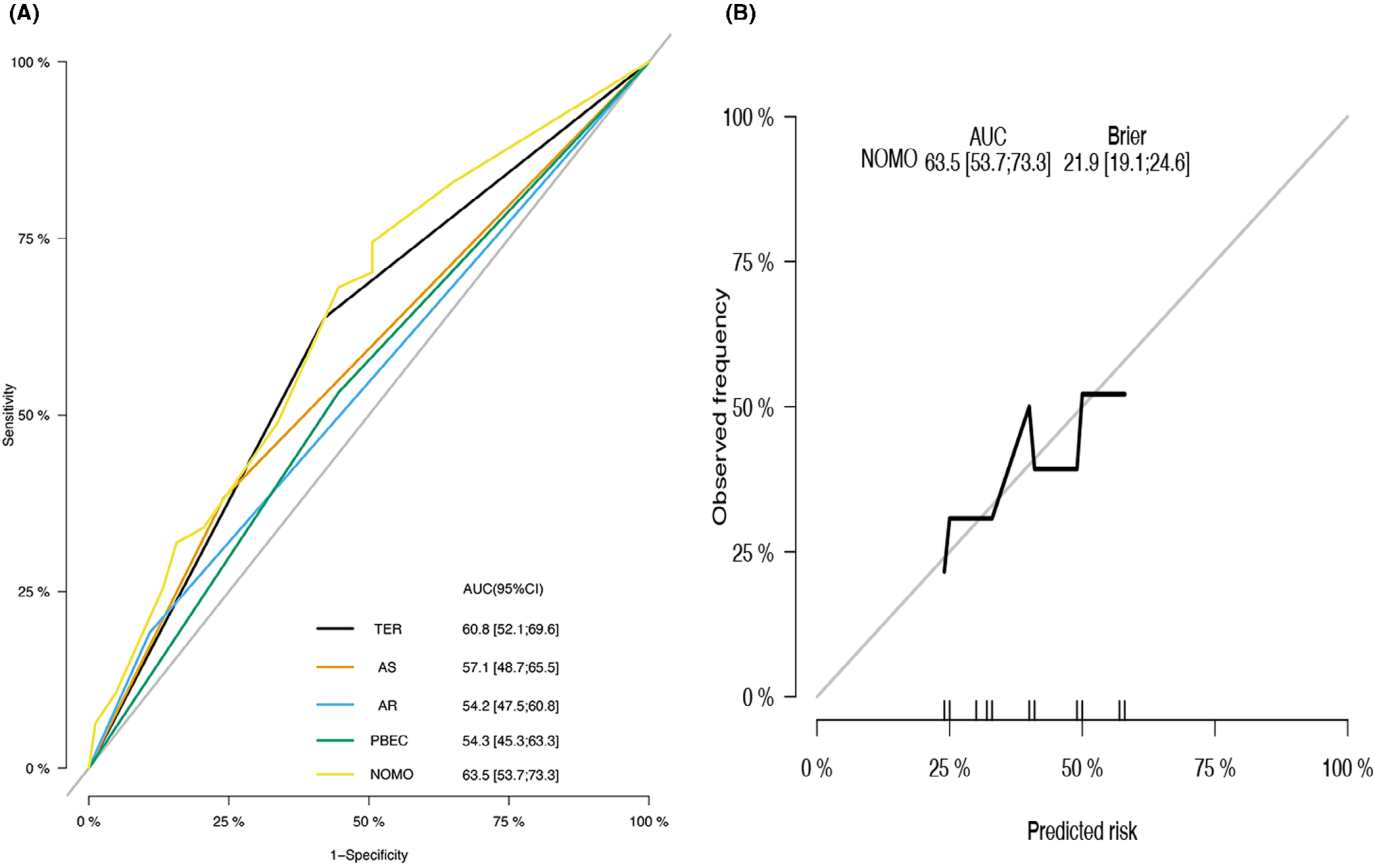
(A) ROC curves of the validation cohort predicting 1‐year probability of uncontrol disease after endoscopic surgery with corresponding AUC values. (B) Calibration in the validation cohort for predicting patient risk of recurrence. The x‐axis is nomogram‐predicted probability of survival and y‐axis is actual survival. The reference line is 45° and indicates perfect calibration. AS, asthma; AUC, area under curve; CI, confidence interval; PBEC, peripheral blood eosinophil count; ROC, receiver operating characteristic; TEN, tissue eosinophil number; TER, tissue eosinophil ratio

Both internal and external validation of the nomogram was performed in this study. The plotted calibration curves correspond to the ideal plot (45°line), which reveals a favourable agreement on the nomogram estimation and the actual observation regarding the probability of uncontrolled disease after the 1 year of post endoscopic sinus surgery. In the training cohort, the nomogram showed the highest accuracy with an AUC of 0.760 (95% confidence interval [CI], 0.688–0.830) (Figure 2A). The corresponding calibration plot indicates the similarity in the estimation made by the nomogram and clinical findings made during the follow‐up period for the recurrence of CRSwNP (Figure 2B). In the validation cohort, the nomogram prediction was 0.635 (95% CI, 0.537–0.733) (Figure 3A). The calibration curve showed a concurrence of predicted probability with the actual probability (Figure 3B).

**FIGURE 4:**
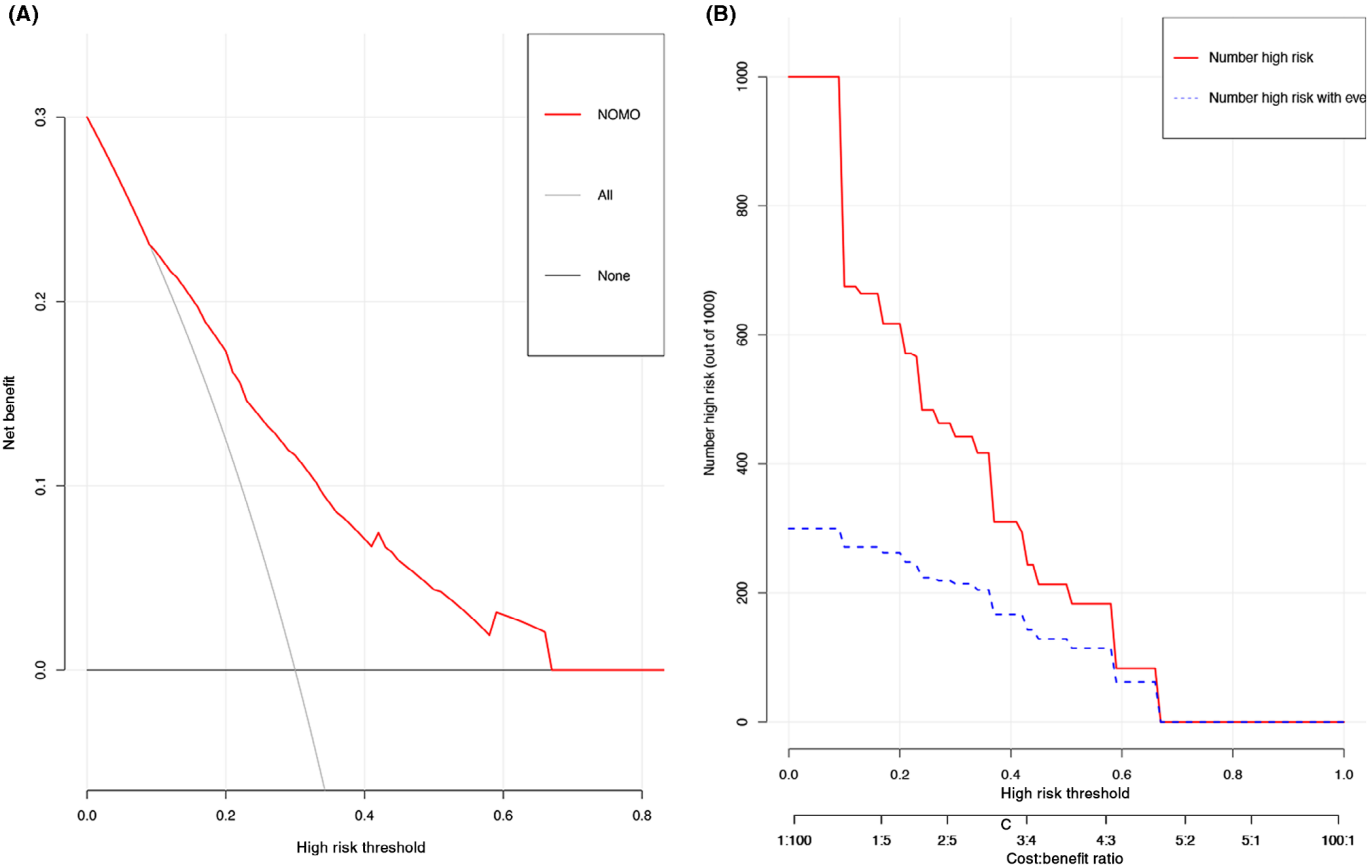
(A) Decision curve analyses in the training cohorts: A perfect prediction model (grey line), screen none (horizontal solid black line) and screen based on the nomogram (blue thick dash line). (B) Clinical impact curve of the nomogram plots the number of CRS patients classified as high risk, and the number of cases classified as high risk with uncontrol disease at each high risk threshold.

To assess the clinical applicability of our risk prediction nomogram, clinical impact curve analysis (CICA) and decision curve analysis (DCA) was also performed. The CICA and DCA visually exhibited that the nomogram had superior practical ranges of threshold probabilities and an overall net benefit in terms of outcome for the impacted patient (Figure 4A,B).

## 4 DISCUSSION

CRS is a group of multifactorial diseases, associated with asthma, allergy, high tissue eosinophil ratio and blood eosinophil counts. CRS is generally treated by pharmacotherapy or by FESS.^9^ In this study, we evaluated CRS patients who had an average follow‐up time of 1 year after undergoing FESS. Clinical characteristics in CRS is very important, as it plays a deciding role in predicting the possibility of postoperative uncontrolled disease in these patients. If patients at a higher risk for revisional surgery, personalised treatments or targeted therapies should also be used to directed the disease control.

### 4.1 Asthma

In 2012, a multicentre study conducted by the Global Allergy and Asthma Network of Excellence (GA^2^LEN) showed that asthma was associated with CRS in all age groups, irrespective of gender and smoking behaviour.^10^ Our group previously reported that extensive endoscopic sinus surgery (EESS) improved the surgery outcomes in CRS with asthma.^11^ In a 12‐year study, asthma was identified as the only factor that increased the chance of recurrence in patients with either CRSwNP or CRSsNP (chronic rhinosinusitis without nasal polyps).^7^, ^12^ Our current study also showed that asthma was the important factor for disease control after surgery, as demonstrated in univariate and multivariate analysis. In the training cohort, the AUC of the asthma models was 0.665 (0.593–0.737). However, CRS with or without asthma is an indisputable element affecting its prognosis.

### 4.2 Allergy

The causal relationship between allergy and CRS is still debatable, however, the risks of CRSwNP are higher in patients with co‐existing allergy and asthma conditions^10^ A population‐based study reported the AR higher prevalence, before the diagnosis of CRSsNP or CRSwNP in comparison with patients without CRS.^13^ A multicentre cross‐sectional study in China reported that many occupational factors are significantly associated with the CRS,^14^ especially exposure to dust or smoke, coal cooking fumes, chemical gases (such as isocyanides), cleaning agents and hair‐care products lead to increased risk.^15^ Allergic asthma and rhinitis caused by inhaled allergens, are mainly elicited by a TH2‐dominated immune response associated with increased serum IgE levels.^16^ Allergy rhinitis with high IgE expression may also affect the disease control of CRSwNP after the surgery. Recently, a randomised phase 3 trials reported that Omalizumab (IgE antibody) significantly improved the clinical, endoscopic and patient‐reported outcomes in refractory CRSwNP.^17^ Therefore, allergic rhinitis was also considered in the prediction model. In our study, the AUC in the training cohort for the AR model was 0.595 (0.52.8–0.66.2), and it also affected the disease control to a certain extent.

### 4.3 System and local eosinophil

The EPOS2020 and several studies reported the cut‐off points for EOS in blood and tissue. We classified the cohort subjects by using 0.3 × 10^9^/L as a cut‐off value for blood EOS counts and 10% for polyp tissue EOS percentages.^2^ The cut‐off point of 10% tissue EOS has been extensively used for differentiating the eosinophilic CRS.^18^ Lou et al. and Nakayama et al. have also demonstrated a strong correlation between polyp recurrence and tissue EOS numbers.^19^, ^20^ Blood EOS can also reflect the prognosis of chronic sinusitis, but its sensitivity is low as compared to the tissue EOS.^21^, ^22^ Our group has reported that the tissue and blood eosinophilia has an additive effect in predicting the risk of poor disease control after at least 1 year of FESS.^23^ This study further demonstrated using multivariate analysis, that the tissue eosinophilia ratio was an independent factor, affecting the disease control after surgery. The analysis revealed that the number of eosinophils in tissues had no significant effect on CRS disease control. However, EPOS 2020 suggests that tissue eosinophils can be considered as nasal polyps eosinophils in case the tissue eosinophils count was more than 10.^24^ In many pieces of literature, tissue eosinophils ratio was still higher than 10% as the cut‐off value to predict the prognosis of chronic sinusitis nasal polyps.^22^ Therefore, we only included tissue eosinophil ratio (TER) in our Nomogram prediction model.

So far, few studies have focused on the various combination factors among asthma (AS), PBEC, TER, AR and disease control. Interestingly, in our study, the combination of AS, AR, TER, PBEC significantly increased the odds ratio for predicting the possibility of uncontrolled and partly controlled disease. To the best of our knowledge, this observation has not been reported in the literature. Therefore, as the potential predictors, we included allergy, asthma, TER and blood EOS counts, among the various demographic factors in our nomograms. For a long, these factors have been recognised to have a significant impact on the disease control of CRS.

This study also had some limitations due to the small cohort size. In addition, childhood‐onset or adult‐onset asthma in CRSwNP were not confirmed. Further, we could not evaluate the relationship between the prognosis of disease the childhood or adult‐onset asthma.

## 5 CONCLUSIONS

We found that TER and AS were the independent factors affecting the prognosis of CRSwNP. In combination with AR, PBEC, TER and AS, the nomogram model exhibited higher accuracy than with tissue eosinophil ratio and asthma alone. The nomogram model provided relatively accurate and visually prediction for disease control in CRS after FESS and served as a reference for the daily diagnosis and treatment.

## AUTHOR CONTRIBUTIONS

*Study design*: Yinyan Lai, Jianbo Shi, Yueqi Sun. *Data collected and analysis*: Lijie Jiang, Kanghua Wang, Zhaoqi Huang, Cong Li, Tengjiao Lin. *Manuscript drafting*: Tengjiao Lin, Yinyan Lai and Lijie Jiang. *Patient follow‐up*: Yifeng Jiang, Wenxiang Gao, Zhiyin Nie, Rui Zheng, Yueqi Sun, Chuxin Chen.

## CONFLICT OF INTEREST

There are no further conflicts of interest to declare for all authors.

### PEER REVIEW

The peer review history for this article is available at https://publons.com/publon/10.1111/coa.13949.

## CONSENT FOR PUBLICATION

Not applicable.

## ETHICS STATEMENT

The study approved by the Ethics Committee of the First Affiliated Hospital of Sun Yat‐sen University ([2017]164).

## Supporting information


**Appendix S1.** Supporting Information

## Data Availability

All data, models and code generated or used during the study appear in the submitted article.
