# Supplementary material for: A novel risk score for disease control prediction of chronic rhinosinusitis
Source: Clin Otolaryngol. 2022 Jun 30;47(5):568–76. doi: 10.1111/coa.13949 (PMC9542583; doi:10.1111/coa.13949)
Supplement: Supplementary file 1 — Appendix S1. Supporting Information [file COA-47-568-s001.docx]

|  | Controlled  (all of the following) | Partly controlled  (at least 1 present) | Uncontrolled  (3 of more features of partly controlled CRS) |
| --- | --- | --- | --- |
| Nasal blockage | Not present or not  bothersome | Present on most days of the week | Present on most days of the week |
| Rhinorrhea/Postnasal drip | Little and mucous | Mucopurulent on most days of the week | Mucopurulent on most days of the week |
| Facial pain/Pressure | Not present or not bothersome | Present on most days of the week | Present on most days of the week |
| Smell | Normal or only slightly impaired | Impaired | Impaired |
| Sleep disturbance or fatigue | Not present | Present | Present |
| Nasal endoscopy | Healthy or almost healthy mucosa | Disease mucosa (nasal polyps, mucopurulent secretions, inflamed mucosa) | Disease mucosa (nasal polyps, mucopurulent secretions, inflamed mucosa) |
| Rescue treatment | Not needed | Need of 1 course of rescue treatment | Symptoms persist despite rescue treatments |

**Table S1. Assessment of current clinical control of CRS（in the last month）**

Symptoms of CRS^1^; For research VAS≤5^2^；For research VAS＞5^3^
